# A breast cancer gene signature for indolent disease

**DOI:** 10.1007/s10549-017-4262-0

**Published:** 2017-04-27

**Authors:** Leonie J. M. J. Delahaye, Caroline A. Drukker, Christa Dreezen, Anke Witteveen, Bob Chan, Mireille Snel, Inès J. Beumer, Rene Bernards, M. William Audeh, Laura J. van’t Veer, Annuska M. Glas

**Affiliations:** 1 Agendia NV, Science Park 406, 1098 XH Amsterdam, The Netherlands; 2 Department of Surgical Oncology and Division of Molecular Carcinogenesis, Netherlands Cancer Institute, PO Box 90203, 1006 BE Amsterdam, The Netherlands; 3 Department of Surgery, Academic Medical Center, University of Amsterdam, PO Box 22660, 1100 DD Amsterdam, The Netherlands; 4 Agendia Inc, 22 Morgan, Irvine, CA 92618 USA; 5 Department of Laboratory Medicine, UCSF Helen Diller Family Comprehensive Cancer Centre, 2340 Sutter Street, San Francisco, CA 94115 USA

**Keywords:** Breast cancer, MammaPrint, Ultralow threshold, Indolent disease

## Abstract

**Purpose:**

Early-stage hormone-receptor positive breast cancer is treated with endocrine therapy and the recommended duration of these treatments has increased over time. While endocrine therapy is considered less of a burden to patients compared to chemotherapy, long-term adherence may be low due to potential adverse side effects as well as compliance fatigue. It is of high clinical utility to identify subgroups of breast cancer patients who may have excellent long-term survival without or with limited duration of endocrine therapy to aid in personalizing endocrine treatment.

**Methods:**

We describe a new ultralow risk threshold for the 70-gene signature (MammaPrint) that identifies a group of breast cancer patients with excellent 20 year, long-term survival prognosis. Tumors of these patients are referred to as “indolent breast cancer.” We used patient series on which we previously established and assessed the 70-gene signature high–low risk threshold.

**Results:**

In an independent validation cohort, we show that patients with indolent breast cancer had 100% breast cancer-specific survival at 15 years of follow-up.

**Conclusions:**

Our data indicate that patients with indolent disease may be candidates for limited treatment with adjuvant endocrine therapy based on their very low risk of distant recurrences or death of breast cancer.

**Electronic supplementary material:**

The online version of this article (doi:10.1007/s10549-017-4262-0) contains supplementary material, which is available to authorized users.

## Introduction

Clinical–pathological-based guidelines, as defined by the Sankt Gallen Consensus Panel and the National Comprehensive Cancer Network, are used to recommend treatment decisions in early-stage breast cancer. These guidelines combine different clinical–pathological factors including age, tumor size, lymph node, grade, and hormonal receptor status to guide the choice of therapy. Recently, genomic information has also been integrated into the treatment decision process [1, 2].

The 70-gene prognosis signature MammaPrint improves the risk assessment of early-stage breast cancer and allows more personalized treatment recommendation for breast cancer patients. This genomic test provides risk of distant recurrence for node-negative and node-positive breast cancer based on its gene expression of the 70-gene signature [3–5]. Our original validation study showed that adjuvantly untreated node-negative patients whose tumors are classified by the 70-gene signature as low risk have a 10-year 92% overall survival (OS), whereas patients classified as high risk by the 70-gene signature have a 59.5% OS [5]. Recently, the prospective randomized trial MINDACT including 6693 early-stage breast cancer patients with node-negative or up to three node-positive disease (EORTC 10041/BIG 03-4; NCT 00433589) confirmed our earlier findings and, in addition, showed for the first time and most importantly prospectively that clinical–pathological high-risk but 70-gene low-risk patients do not gain a clinically meaningful benefit from adjuvant chemotherapy [6]. This provided the highest level of evidence for the clinical utility of the 70-gene signature low-risk patients who thus can safely forego chemotherapy, even in the clinical–pathological high-risk setting, including patients with node-positive disease. Furthermore, the MINDACT trial revealed an excellent 97.8% distant recurrence-free interval at 5 years for patients with node-negative, hormone-receptor positive, HER2-negative, 70-gene low-risk tumors, who were untreated with chemotherapy [6].

Our previous studies have showed that screen-detected breast cancers are associated with a higher likelihood of a 70-gene low-risk signature [7, 8]. This patient population is increasing given the higher compliance to mammographic screening in most countries. In addition, we also showed that a large proportion of these low-risk tumors were at ‘ultralow’ risk for developing distant metastases [7, 8]. This ultralow risk category was previously defined on patients’ 5-year outcome data [3]. The recent publication of our Netherlands Cancer Institute NKI295 breast cancer series with 18.5-year median follow-up [9], allowed us to reset our ultralow risk threshold to establish an ultralow risk group with very long-term indolent disease, and is reported here. Such an indolent disease group with prolonged excellent outcome would guide patients’ need for limited, or extended endocrine therapy.

## Patients and methods

### Study population

Three patient cohorts were used for determination, locking, and validation of the long-term ultralow risk threshold. These cohorts were previously described and 70-gene signature results were available, “NKI295” [4, 9], “Transbig” [5], and “RASTER” [10, 11].

From the first cohort, “NKI295,” age under 55, only node-negative patients were selected resulting in 151 patients’ samples of whom 96% did not receive adjuvant chemotherapy, and 93% did not receive any systemic treatment, following Dutch national guidelines at the time [4]. This patient cohort was used for determination and locking of the ultralow risk threshold (patient characteristics, see Supplementary Information, Table 1) [4]. Study design, patient eligibility, study logistics, and clinical–pathological parameters of the study have been described elsewhere [4, 9]. The median follow-up of this cohort was 18.5 years.

The second cohort, “Transbig,” age under 61, was used for validation of the ultralow risk threshold. Details on study design and clinical parameters of this cohort were previously described [5]. All 302 samples were used. Patients were all systemically untreated. The median follow-up of this cohort was 13.6 years (patient characteristics, see Supplementary Information, Table 2).

The third cohort, “RASTER,” originates from the RASTER clinical study, age under 61, which is a prospective observational study conducted in accordance with Dutch CBO 2004 practice guidelines [10, 11]. Primarily, in this study, we used this dataset for the technical transfer of the ultralow threshold to the diagnostic FDA-cleared MammaPrint formalin-fixed paraffin-embedded (FFPE) test. Extensive description of the RASTER study design, patient eligibility, and study logistics of the included patients can be found elsewhere (patient characteristics, see Supplementary Information, Table 3 [10]). In this study, 345 RASTER patients were included of whom both fresh and FFPE sample materials were available. The median follow-up of this cohort was 5 years [11].

The original studies followed REMARK criteria [12].

An additional patient cohort was used to estimate the size of the indolent group in contemporary clinical practice within the early-stage breast cancer population. This cohort is a 2014–2015 US and Europe diagnostic dataset of 13,794 patients tested for MammaPrint on FFPE samples.

### Sample processing and 70-gene signature index

Sample processing and generation of the 70-gene signature indices was performed at the time of the original studies or diagnostic assessment [4, 5, 10].

**Fig. 1:**
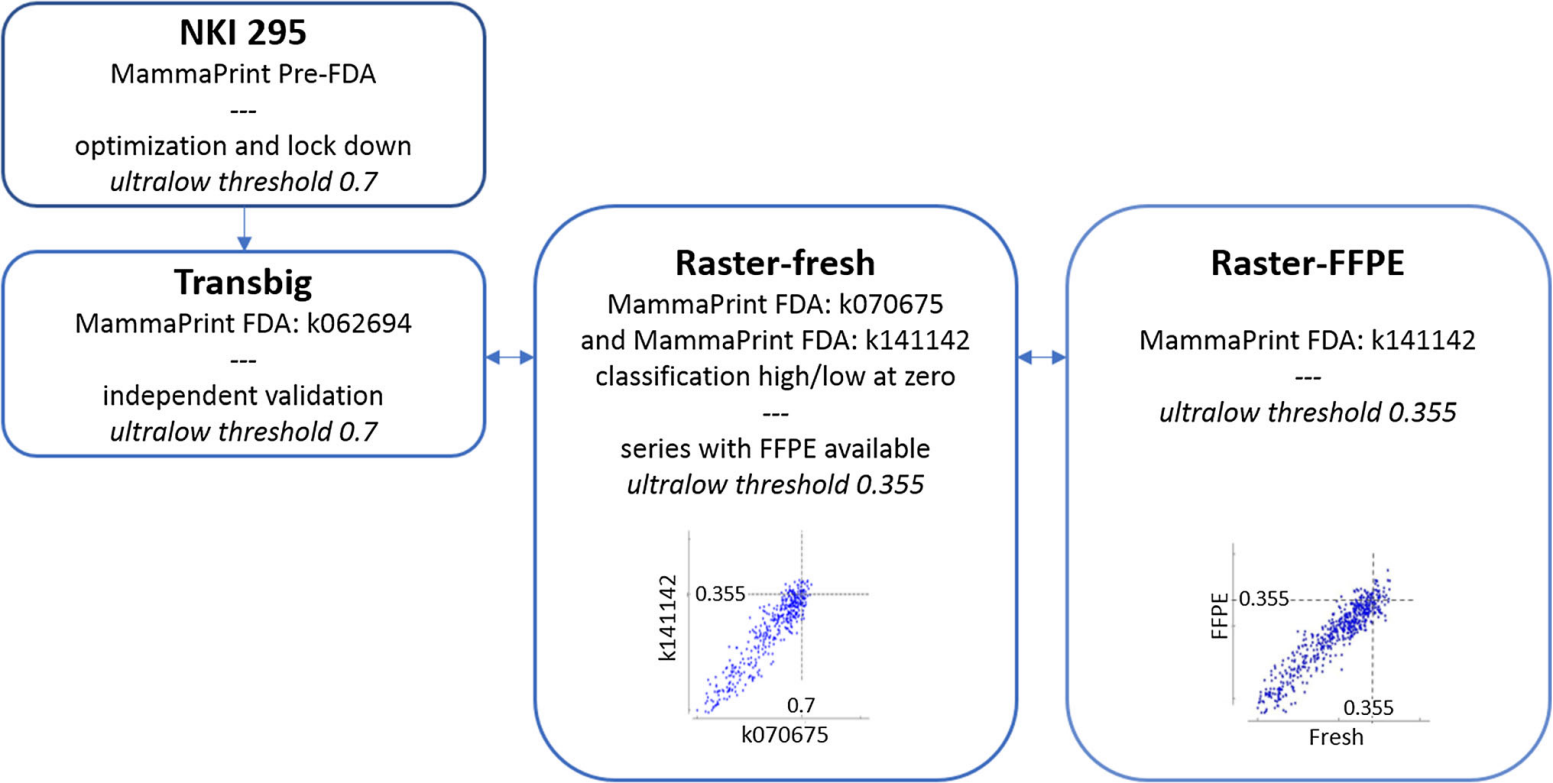
Overview of different steps taken to establish, lock, validate, and convert the ultralow risk threshold fresh tissue to current diagnostic MammaPrint for FFPE tissue. Scatterplots are given for 70-gene signature indices across different FDA-cleared versions with indication of the accompanying 70-gene signature ultralow risk thresholds

The 70-gene signature underwent technical advances over time, was made available for fresh frozen, fresh-RNA retain as well as FFPE preserved tissue, which each were authenticated in technical updates and/or FDA clearances [13]. In this study, we spanned the technical advances, and transferred the ultralow risk threshold across the different versions of the 70-gene signature test and datasets used, up to the current MammaPrint FFPE diagnostic test (Fig. 1).

### Statistical analysis

Kaplan–Meier analysis was performed for the 70-gene signature classifications (ultralow, low, and high risk). This survival analysis was based on breast cancer-specific survival (BCSS), where BCSS was defined as the time from surgery until breast cancer-related death. All analyses were performed using *SPSS version 22.0.*

## Results

Within the 70-gene signature low-risk population, we aimed to identify a sub-group of breast cancer patients who have indolent disease with excellent long-term survival. In this study, we set out to establish the classification threshold for indolent disease, as defined by a 100% BCSS at 20 years of follow-up. Further validation of the threshold was performed. Details of the steps taken to generate an indolent classification threshold that is applicable to the current diagnostic test are outlined in Fig. 1 and described in the following paragraphs.

### Indolent classification threshold

**Fig. 2:**
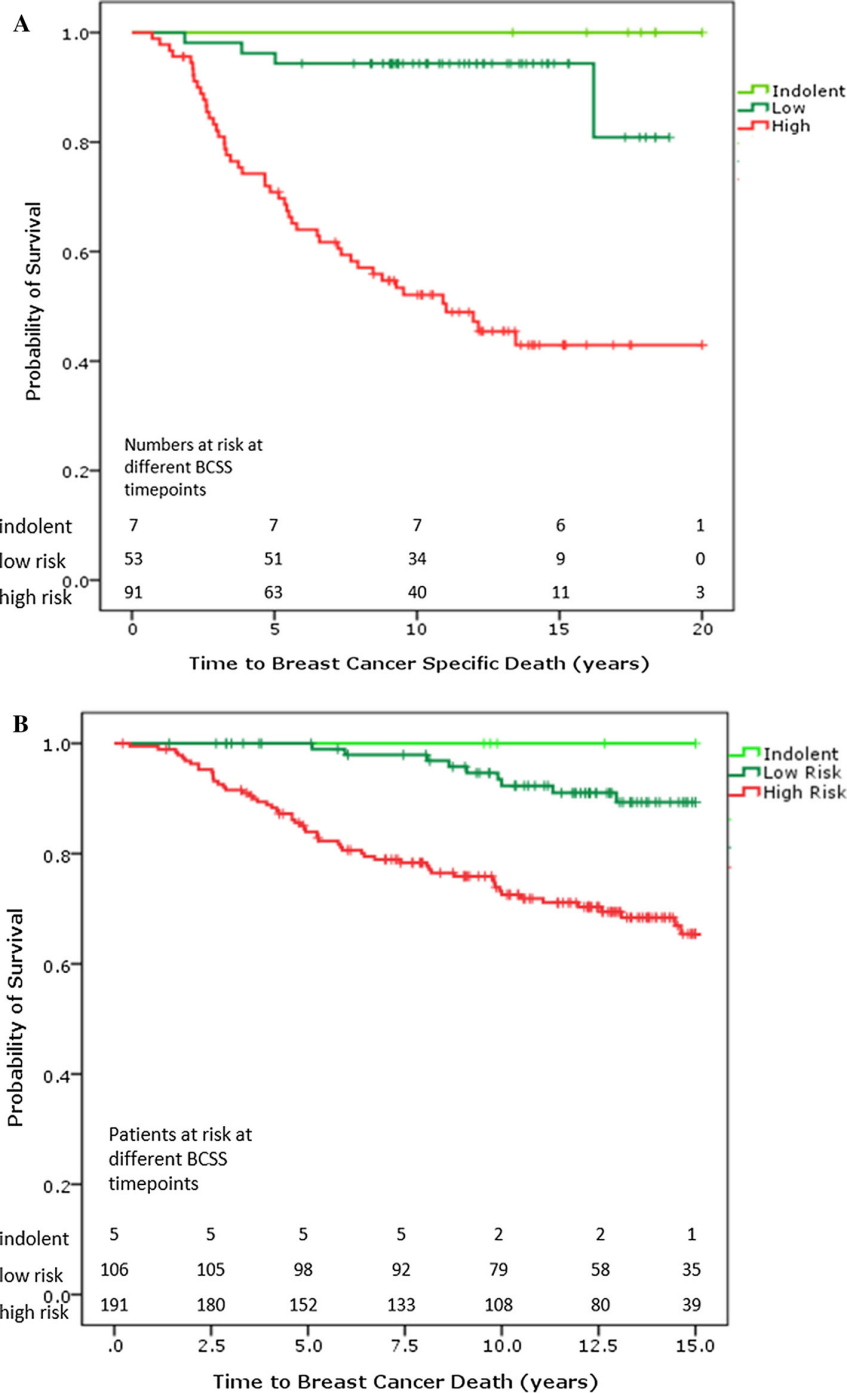
**a** Breast cancer-specific survival (BCSS) of all node-negative patients (*n* = 151) from NKI295 by 70-gene signature indolent, low-, and high-risk classification. **b** Breast cancer-specific survival (BCSS) of Transbig by 70-gene signature indolent, low-, and high-risk classification

Initially, we established a threshold for indolent disease using the 5-year follow-up data of the 78 patient series on which we had developed the 70-gene signature [7, 8]. We now revisited the threshold to establish a true indolent classification threshold based on BCSS at 20 years of follow-up of the NKI295 series (Figs. 1, 2a; Table 1). We used the lymph node-negative population of the NKI295 cohort (*n* = 151), which has a median follow-up of 18.5 years as previously described [9]. Optimization of the ultralow risk threshold based on the longer follow-up of this dataset resulted in a threshold change from the previously published 0.6 to 0.7, which was subsequently locked. This stringency ensures identification of an indolent patient group with 100% survival for the complete follow-up period.

### Independent clinical validation of the indolent classification threshold

The threshold locked at 0.7 was then independently validated in the Transbig breast cancer cohort [5], a node-negative and adjuvantly untreated patient series (*n* = 302) with median follow-up of 13.6 years. The additional advantage of this dataset is that it was processed using the MammaPrint FDA-cleared version k062694 for fresh frozen sample (Fig. 1) [13, 14]. Kaplan–Meier survival analysis for indolent, low-, and high-risk patients up to 15 years revealed a 100% BCSS, providing an independent validation of the threshold (Fig. 2b). Five patients (1.7%) were classified as indolent, 106 patients (35.1%) were classified as low risk with a survival rate of 89.3% (82.6–96%), and the remaining 191 patients (63.2%) were classified as high risk with a 15-year BCSS of 65.5% (57.5–73.5%) (Fig. 2b; Table 1).

### Indolent classification threshold for FFPE tissue

The 70-gene signature was over time further refined by introducing microarray single-channel hybridization and providing a 70-gene signature index based on a sample’s correlation to a predefined high-risk and low-risk standardized value [3, 13, 15]. All steps were defined in FDA technical updates and clearances [13]. In addition, the 70-gene signature high-low classification threshold was repositioned to zero for ease of interpretation, and then also became available for FFPE tissue [13]. To parallel these changes for the definition of the ultralow risk threshold, the RASTER dataset was used where three sets of MammaPrint indices were available: the original publication [10], FDA k070675-fresh-RNA retain, as well as the current versions of MammaPrint fresh and FFPE [13], FDA k141142-FFPE (Fig. 1). Using linear regression, the ultralow threshold of 0.7 in FDA k070675, became 0.355 (regression equation *y* = 1.11*x* + 0.022), after repositioning the high/low threshold at zero, FDA k141142. For FFPE tissue, the ultralow threshold stays at 0.355 (Fig. 1).

### Prevalence of indolent disease in early-stage breast cancer

To get an estimate of the prevalence of the indolent group, we compared the percentage of patients classified as indolent in each of the cohorts that were used in this study as well as in a diagnostic dataset that was subjected to MammaPrint testing in 2014–2015 (Table 1). The percentage of indolent patients is lower in the NKI295 and Transbig studies compared to RASTER and the diagnostic dataset. The NKI295 and Transbig datasets include patients diagnosed in earlier years than the RASTER and diagnostic dataset, and are therefore less influenced by the increase of screen-detected tumors.

**Table 1:** Percentage of identified indolent, low-, and high-risk patients across different datasets

Datasets		Ultralow risk threshold	Total	Percentage of patients per 70-gene signature classification group		
				Indolent (%)	Low risk^a^ (%)	High risk (%)
NKI295	Establish threshold and locked	0.7	*n* = 151 (only LN−) Age <55	4.6% (*n* = 7)	35.1% (*n* = 53)	60.3% (*n* = 91)
Transbig	Independent validation	0.7	*n* = 302 Age <61	1.7% (*n* = 5)	35.1% (*n* = 106)	63.2% (*n* = 191)
RASTER FFPE	Validation FFPE	0.355	*n* = 345 Age <61	11.9% (*n* = 41)	40.0% (*n* = 138)	48.1% (*n* = 166)
Diagnostics: MammaPrint FFPE 2014–2015	Review in diagnostics (FFPE)	0.355	*n* = 13,794 All ages	12.3% (*n* = 1701)	40.4% (*n* = 5573)	47.3% (*n* = 6620)

^a^Not indolent

## Discussion

We defined and validated an ultralow risk threshold of the 70-gene signature test which identifies patients with a tumor of indolent clinical course who have excellent long-term survival. We earlier established an indolent disease threshold but that threshold was based on only 5-year follow-up of the original 78 patients on which we developed the 70-gene signature [7, 8]. Recently, however, we updated the follow-up of this series and the original validation series (NKI295) to 25 years [9], and we used this latter series to optimize and lock an ultralow threshold for long-term indolent disease (Fig. 1a). In addition, we performed an independent validation of this new ultralow threshold in the Transbig series (Fig. 2b) [5]. Finally, the clinically validated ultralow threshold was transferred to the current FDA-cleared MammaPrint FFPE version FDA k141122, to allow use in current diagnostics (Fig. 1).

Our independent validation of the ultralow risk threshold in the systemically untreated Transbig cohort (*n* = 302) shows 100% BCSS at 15 years of follow-up for this group. With a BCSS of 100%, there is no achievable gain in survival by providing systemic treatment to this group. In fact, side effects of adjuvant treatment may negatively affect long-term survival rather than provide benefit. Since the proportion of indolent disease patients in these older series with long-term follow-up is low, a larger series would be needed to further establish clinical utility.

Interestingly, the number of patients in the indolent group varies between the different studies. The NKI295 series only had a small percentage of lymph node-negative indolent disease patients, whereas the percentage increases to >12% in the more recently diagnosed RASTER series where a large proportion of cancers were mammographically screen-detected, a feature we previously established to be associated with 70-gene ultralow risk breast cancers (Table 1) [7, 8]. Of note, the different series used have different average ages of diagnosis: their median age varies from a relatively young population to the contemporary series where median age is at least a decade older, which also influences the proportion of ultralow risk (Supplementary Tables 1, 2, 3). Interestingly, the current diagnostic series of 13,794 patients even includes a considerable proportion of node-positive patients. We anticipate that post-menopausal older patients who have screen-detected, node negative, hormone-receptor positive disease may have a larger proportion of indolent disease.

The ultralow threshold identifying patients with no death of breast cancer up to 20 years after diagnosis may provide guidance to personalize endocrine treatment for women diagnosed with early breast cancer, and given it identifies an indolent disease patient cohort, could aid in reducing overtreatment.

## Electronic supplementary material

Below is the link to the electronic supplementary material.

Supplementary material 1 (DOCX 64 kb)
