# Supplementary material for: A breast cancer gene signature for indolent disease
Source: Breast Cancer Res Treat. 2017 Apr 27;164(2):461–6. doi: 10.1007/s10549-017-4262-0 (PMC5487706; doi:10.1007/s10549-017-4262-0)
Supplement: Supplementary file 1 — Supplementary material 1 (DOCX 64 kb) [file 10549_2017_4262_MOESM1_ESM.docx]

# Supplementary information

**Supplementary Table 1:** NKI295 – characteristics for 151 node negative patients [4]

|  |  |  |  | **70-gene signature** | | | | | |  |
| --- | --- | --- | --- | --- | --- | --- | --- | --- | --- | --- |
|  |  | **Total** | | **Indolent** | | **Low** | | **High** | | **p-value** |
| **Variable** |  | **N=151** | **%** | **N=7** | **4.6%** | **N=53** | **35.1%** | **N=91** | **60.3%** |  |
| Age | <40 | 36 | 23.8 | 1 | 14.3 | 4 | 7.5 | 31 | 34.0 | 0.001 |
|  | 40-44 | 42 | 27.8 | 5 | 71.4 | 17 | 32.1 | 20 | 22.0 |  |
|  | 45-49 | 49 | 32.5 | 0 | 0.0 | 24 | 45.3 | 25 | 27.5 |  |
|  | 50-54 | 24 | 15.9 | 1 | 14.3 | 8 | 15.1 | 15 | 16.5 |  |
| Surgery | Mastectomy | 61 | 40.4 | 4 | 57.1 | 20 | 37.7 | 37 | 40.7 | 0.615 |
|  | BCS | 90 | 59.6 | 3 | 42.9 | 33 | 62.3 | 54 | 59.3 |  |
| Tumorsize | Median | 20.0 | | 18.0 | | 18.0 | | 22.0 | | 0.074 |
| pT | 1 | 82 | 54.3 | 5 | 71.4 | 34 | 64.2 | 43 | 47.3 | 0.094 |
|  | 2 | 69 | 45.7 | 2 | 28.6 | 19 | 35.8 | 48 | 52.7 |  |
|  | 3 | - | - | - | - | - | - | - | - |  |
| Grade | 1 | 34 | 22.5 | 4 | 57.1 | 23 | 43.4 | 7 | 7.7 | <0.001 |
|  | 2 | 46 | 30.5 | 2 | 28.6 | 21 | 39.6 | 23 | 25.3 |  |
|  | 3 | 71 | 47.0 | 1 | 14.3 | 9 | 17.0 | 61 | 67.0 |  |
| ER | Neg | 42 | 27.8 | 0 | 0.0 | 2 | 3.8 | 40 | 44.0 | <0.001 |
|  | Pos | 109 | 72.2 | 7 | 100.0 | 51 | 96.2 | 51 | 56.0 |  |
| ET* | No | 145 | 96.0 | 7 | 100.0 | 50 | 94.3 | 88 | 96.7 | 0.672 |
|  | Yes | 6 | 4.0 | 0 | 0.0 | 3 | 5.7 | 3 | 3.3 |  |
| CT* | No | 145 | 96.0 | 7 | 100.0 | 51 | 96.2 | 87 | 95.6 | 0.845 |
|  | Yes | 6 | 4.0 | 0 | 0.0 | 2 | 3.8 | 4 | 4.4 |  |

* 141 patients received no endocrine and no chemotherapy

**Supplementary Table 2:** TRANSBIG – patient characteristics [5]

|  |  |  |  | **70-gene signature** | | | | | |  |
| --- | --- | --- | --- | --- | --- | --- | --- | --- | --- | --- |
|  |  | **Total** |  | **Indolent** | | **Low** | | **High** | | **p-value** |
| **Variable** |  | **N=302** | **%** | **N=5** | **1.7%** | **N=106** | **35.1%** | **N=191** | **63.2%** |  |
| Age | <40 | 49 | 16.2 | 1 | 20.0 | 14 | 13.2 | 34 | 17.8 | 0.404 |
|  | 40-44 | 65 | 21.5 | 1 | 20.0 | 21 | 19.8 | 43 | 22.5 |  |
|  | 45-49 | 76 | 25.2 | 3 | 60.0 | 29 | 27.4 | 44 | 23.0 |  |
|  | 50-60 | 112 | 37.1 | 0 | 0.0 | 42 | 39.6 | 70 | 36.6 |  |
| Surgery | Mastectomy | 85 | 28.1 | 1 | 20.0 | 27 | 25.5 | 57 | 29.8 | 0.667 |
|  | BCS | 217 | 71.9 | 4 | 80.0 | 79 | 74.5 | 134 | 70.2 |  |
| Tumorsize | Median | 20.0 | | 20.0 | | 20.0 | | 21.0 | | 0.009 |
| pT | 1 | 110 | 36.4 | 2 | 40.0 | 50 | 47.2 | 58 | 30.4 | 0.068 |
|  | 2 | 191 | 63.2 | 3 | 60.0 | 56 | 52.8 | 132 | 69.1 |  |
|  | 3 | 1 | 0.3 | 0 | 0.0 | 0 | 0.0 | 1 | 0.5 |  |
| Grade* | 1 | 47 | 15.9 | 1 | 20.0 | 28 | 26.7 | 18 | 9.7 | <0.001 |
|  | 2 | 125 | 42.2 | 2 | 40.0 | 68 | 64.8 | 55 | 29.6 |  |
|  | 3 | 124 | 41.9 | 2 | 40.0 | 9 | 8.6 | 113 | 60.8 |  |
| ER | Neg | 90 | 29.8 | 0 | 0.0 | 5 | 4.7 | 85 | 44.5 | <0.000 |
|  | Pos | 212 | 70.2 | 5 | 100.0 | 101 | 95.3 | 106 | 55.5 |  |
| ET | No | 302 | 100.0 | 5 | 100.0 | 106 | 100.0 | 191 | 100.0 |  |
|  | Yes | 0 | 0.0 | 0 | 0.0 | 0 | 0.0 | 0 | 0.0 |  |
| CT | No | 302 | 100.0 | 5 | 100.0 | 106 | 100.0 | 191 | 100.0 |  |
|  | Yes | 0 | 0.0 | 0 | 0.0 | 0 | 0.0 | 0 | 0.0 |  |

*6 missing

**Supplementary Table 3:** RASTER – patient characteristics [10, 11]

|  |  |  |  | **70-gene signature** | | | | | |  |
| --- | --- | --- | --- | --- | --- | --- | --- | --- | --- | --- |
|  |  | **Total** |  | **Indolent** | | **Low** | | **High** | | **p-value** |
| **Variable** |  | **N=345** | **%** | **N=41** | **12%** | **N=138** | **40%** | **N=166** | **48%** |  |
| Age | <40 | 48 | 13.9 | 3 | 7.3 | 8 | 5.8 | 37 | 22.3 | <0.001 |
|  | 40-44 | 64 | 18.6 | 5 | 12.2 | 26 | 18.8 | 33 | 19.9 |  |
|  | 45-49 | 91 | 26.4 | 12 | 29.3 | 34 | 24.6 | 45 | 27.1 |  |
|  | 50-60 | 142 | 41.2 | 21 | 51.2 | 70 | 50.7 | 51 | 30.7 |  |
| Surgery | Mastectomy | 64 | 18.6 | 7 | 17.1 | 23 | 16.7 | 34 | 20.5 | 0.673 |
|  | BCS | 281 | 81.4 | 34 | 82.9 | 115 | 83.3 | 132 | 79.5 |  |
| Tumorsize | Median | 17.0 | | 16.0 | | 14.5 | | 19.5 | | <0.001 |
| pT | 1 | 232 | 67.2 | 33 | 80.5 | 110 | 79.7 | 89 | 53.6 | <0.001 |
|  | 2 | 112 | 32.6 | 8 | 19.5 | 27 | 19.6 | 77 | 46.4 |  |
|  | 3 | 1 | 0.3 | 0 | 0.0 | 1 | 0.7 | 0 | 0.0 |  |
| Grade | 1 | 69 | 20.1 | 14 | 34.1 | 47 | 34.1 | 8 | 4.8 | <0.001 |
|  | 2 | 166 | 48.1 | 26 | 63.4 | 83 | 60.1 | 57 | 34.3 |  |
|  | 3 | 110 | 31.9 | 1 | 2.4 | 8 | 5.8 | 101 | 60.8 |  |
| ER | Neg | 73 | 21.2 | 0 | 0.0 | 2 | 1.4 | 71 | 42.8 | <0.001 |
|  | Pos | 272 | 78.8 | 41 | 100.0 | 136 | 98.6 | 95 | 57.2 |  |
| ET | No | 196 | 56.8 | 29 | 70.7 | 80 | 58.0 | 87 | 52.4 | 0.099 |
|  | Yes | 149 | 43.2 | 12 | 29.3 | 58 | 42.0 | 79 | 47.6 |  |
| CT | No | 174 | 50.4 | 35 | 85.4 | 100 | 72.5 | 39 | 23.5 | <0.001 |
|  | Yes | 171 | 49.6 | 6 | 14.6 | 38 | 27.5 | 127 | 76.5 |  |
